# Investigating the Spectrum of Biological Activity of Ring-Substituted Salicylanilides and Carbamoylphenylcarbamates

**DOI:** 10.3390/molecules15118122

**Published:** 2010-11-10

**Authors:** Jan Otevrel, Zuzana Mandelova, Matus Pesko, Jiahui Guo, Katarina Kralova, Frantisek Sersen, Marcela Vejsova, Danuta S. Kalinowski, Zaklina Kovacevic, Aidan Coffey, Jozef Csollei, Des R. Richardson, Josef Jampilek

**Affiliations:** 1 Department of Chemical Drugs, Faculty of Pharmacy, University of Veterinary and Pharmaceutical Sciences, Palackeho 1/3, 61242 Brno, Czech Republic; 2 Zentiva k.s., U kabelovny 130, 102 37 Prague, Czech Republic; 3 Department of Ecosozology and Physiotactics, Faculty of Natural Sciences, Comenius University, Mlynska dolina Ch-2, 842 15 Bratislava, Slovakia; 4 Department of Biological Sciences, Cork Institute of Technology, Bishopstown, Cork, Ireland; 5 Institute of Chemistry, Faculty of Natural Sciences, Comenius University, Mlynska dolina Ch-2, 842 15 Bratislava, Slovakia; 6 Department of Biological and Medical Sciences, Faculty of Pharmacy in Hradec Kralove, Charles University in Prague, Heyrovskeho 1203, 500 05 Hradec Kralove, Czech Republic; 7 Department of Pathology and Bosch Institute, University of Sydney, Sydney, New South Wales 2006, Australia

**Keywords:** salicylanilides, lipophilicity, EPR study, photosynthetic electron transport inhibition, spinach chloroplasts, *in vitro* anti-fungal activity, *in vitro* anti-bacterial activity, *in vitro* anti-mycobacterial activity, anti-proliferative activity

## Abstract

In this study, a series of twelve ring-substituted salicylanilides and carbamoylphenylcarbamates were prepared and characterized. The compounds were analyzed using RP-HPLC to determine lipophilicity. They were tested for their activity related to the inhibition of photosynthetic electron transport (PET) in spinach (*Spinacia oleracea* L.) chloroplasts. Moreover, their site of action in the photosynthetic apparatus was determined. Primary *in vitro* screening of the synthesized compounds was also performed against mycobacterial, bacterial and fungal strains. Several compounds showed biological activity comparable with or higher than the standards 3-(3,4-dichlorophenyl)-1,1-dimethylurea, isoniazid, penicillin G, ciprofloxacin or fluconazole. The most active compounds showed minimal anti-proliferative activity against human cells in culture, indicating they would have low cytotoxicity. For all compounds, the relationships between lipophilicity and the chemical structure are discussed.

## 1. Introduction

Salicylanilides are an important class of aromatic compounds with a wide range of pharmacological activities. A number of them show anti-bacterial [1,2,3,4], anti-mycobacterial [5,6], anti-fungal [5,7] and anti-protozoal/molluscicidal [8] as well as anti-inflammatory [9,10] or anti-neoplastic activities [11,12,13]. Recently, a number of organic carbamates have been found to be potential anti-bacterial, anti-mycobacterial and anti-viral agents [14,15]. The carbamate residue present in these new molecules contributes as a core component [16] or, incorporated into a known molecule, contributes to the improvement of its pharmacodynamic and pharmacokinetic properties [17]. In particular, the carbamate group was successfully used to protect phenolic drugs [18].

The presence of an amide or thioamide (-NHCO- or -NHCS-) group is characteristic of a number of herbicides acting as photosynthesis inhibitors (acylanilides, thioacylanilides, phenylcarbamates, ureas, *etc.*), e.g., [19,20,21,22]. The wide spectrum of biological effects of salicylanilides includes herbicidal activity [23,24] and they were also found to be uncouplers of photosynthetic phosphorylation [25].

The inhibitory activity of the R’ substituted salicylanilides related to the inhibition of oxygen evolution in spinach chloroplasts was correlated with the hydrophobic parameter π^-^ and the Hammett constant (σ) of the R’ substituents [19,20,21,22,23,24]. These compounds were additionally found to interact with the intermediate D^•^ (Tyr_D_) in the photosynthetic apparatus of spinach chloroplasts [23]. Intermediate D^•^ is situated at the 161^st^ position in the D_2_ protein occurring at the donor side of photosystem (PS) 2. In the presence of R’ substituted salicylanilides, the decreased intensity of the fluorescence emission band at 686 nm (belonging to the chlorophyll-protein complexes mainly in PS 2 [26]) suggested PS 2 as the site of action of the studied inhibitors.

Upon addition of 1,5-diphenylcarbazide (DPC, an artificial electron donor of PS 2 with a known site of action in the intermediate Z^•^/D^•^ on the donor side of PS 2 [27]) to chloroplasts treated with R’ substituted salicylanilides, the inhibition of the oxygen evolution rate was completely restored. This indicated that the core of PS 2 (P 680) and a part of the electron transport chain, at least up to plastoquinone, remained intact. These results are in accordance with those obtained for 3-bromo- and 3,5-dibromosalicylanilides [28] and 3-methylsalicylanilides [24]. However, it should be stressed that 3,5-dibromosalicylanilides with R’ = 3-F and R = 3-Cl interacted not only with the intermediate D^•^ (Tyr_D_), but also with the Z^•^ intermediate (Tyr_Z_), which is situated in the 161^st^ position of the D1 protein occurring at the donor side of PS 2 [28].

The R’-substituted salicylanilides and their 5-nitro, 5-fluoro and 5-bromo analogues also reduced chlorophyll production in suspensions of *Chlorella vulgaris*. Indeed, the anti-algal activity of the studied compounds depended on their lipophilicity and the electronic parameter σ of the R’ substituent [29]. Reduction of the chlorophyll content in *Chlorella vulgaris* was also observed in the presence of 3-methylsalicylanilides [30].

Following on from our previous investigations, [31,32,33,34,35,36,37,38], we examined the syntheses and in particular the herbicidal activity of various aromatic ring-substituted like-carboxamide derivatives. The compounds were tested for their photosynthesis-inhibiting activity (the inhibition of photosynthetic electron transport) in spinach chloroplasts (*Spinacia oleracea* L.) and their site of action in the photosynthetic apparatus was determined. Relationships between the structure of the new compounds and their *in vitro* anti-microbial activities or/and inhibitory activity of photosynthetic electron transport (PET) in spinach chloroplasts are discussed. The compounds were also assessed for activity against various bacterial, mycobacterial and fungal strains. Salicylic derivatives are known as strong chelators of the essential nutrient iron that is needed for cellular proliferation [39,40]. Therefore, the compounds were evaluated as potential iron chelating agents with possible anti-proliferative activity against neoplastic cells.

## 2. Results and Discussion

### 2.1. Chemistry

**Scheme 1:**
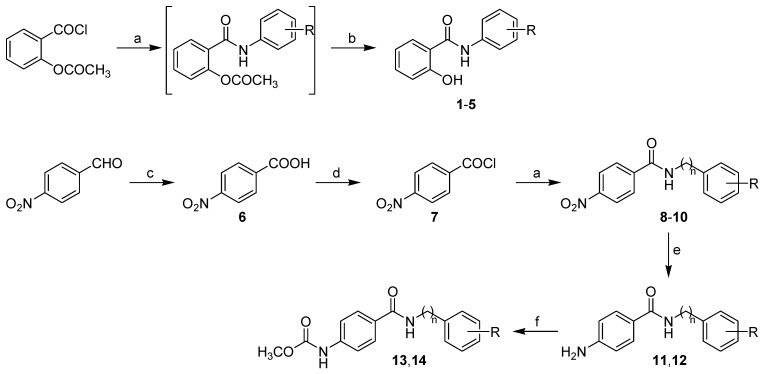
Synthesis of the studied compounds.

The synthetic routes used are shown in Scheme 1. Condensation of commercially available 2-(chlorocarbonyl)phenyl acetate with ring-substituted anilines [32] and subsequent hydrolysis [41] yielded a series of salicylic acid anilides **1**-**5**.

4-Nitrobenzaldehyde was oxidized using KMnO_4_ [42] to yield 4-nitrobenzoic acid (**6**) that was chlorinated with SOCl_2_ and 4-nitrobenzoyl chloride (**7**) was thus obtained [32]. The chloride **7** was condensed with ring-substituted amines, as described previously [32], to afford amides **8**-**10**. Nitrobenzamides **9** and **10** were hydrogenated over activated Raney Ni (Ra-Ni) [43,44] to give aminobenzamides **11** and **12**. Methylphenylcarbamates **13** and **14** were generated by means of the reaction of aminobenzamides **11** and **12** with methyl chloroformiate [45]. 

### 2.2. Lipophilicity

Many low molecular weight drugs cross biological membranes through passive transport, which strongly depends on their lipophilicity. Lipophilicity is a property that has a major effect on absorption, distribution, metabolism, excretion, and toxicity (ADME/Tox) properties as well as pharmacological activity. Lipophilicity has been studied and applied as an important drug property for decades [46].

**Figure 1:**
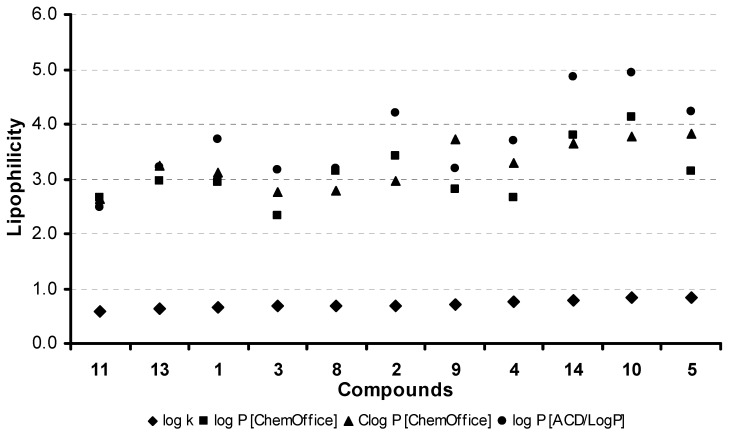
Comparison of the log *P* data calculated using the two programs with the experimentally found log *k* values. The compounds are arranged in the ascending manner according to the experimental log *k* values.

Hydrophobicities (log *P*/Clog *P*) of the compounds **1**-**5** and **8**-**14** were calculated using two commercially available programs (ChemDraw Ultra 10.0 and ACD/LogP) and also measured by means of the RP-HPLC determination of capacity factors *k* with subsequent calculation of log *k*. The procedure was performed under isocratic conditions with methanol as an organic modifier in the mobile phase using an end-capped non-polar C_18_ stationary RP column. The results are shown in Table 1 and illustrated in Figure 1.

The results obtained with all the compounds show that the experimentally-determined lipophilicities (log *k*) of compounds **1**-**5** and **8**-**11**, **13**, **14** are lower than those indicated by the calculated log *P*/Clog *P*, as shown in Figure 1. The results indicate that experimentally-determined log *k* values correlate relatively poorly with the calculated values (see Table 1 or Figure 1), which is probably caused by intramolecular interactions of these highly functionalized molecules.

As expected, aminobenzamide **11** showed the lowest lipophilicity (log *k*), while compounds **5** and **10** exhibited the highest log *k* values. Generally, nitro compounds **8**-**10** showed relatively high lipophilicity in comparison with amino derivatives, e.g. **11** < **13** < **9**. Within the series of ring-substituted salicylanilides **1**-**5**, the determined log *k* values corresponded to the expected lipophilicity increase **1** (2-CH_3_) < **3** (2-OCH_3_) < **2** (2,6-CH_3_) < **4** (2-OC_2_H_5_) < **5** (2-OC_3_H_7_). This dependence is approximately linear and thus, it can be assumed that log *k* values specify lipophilicity within the individual series of the studied compounds more precisely than calculated log *P*/Clog *P* data.

### 2.3. Biological activities

The compounds under investigation could be divided into three groups based on their chemical structure: Group 1 included salicylanilides **1**-**5**; Group 2 contained nitro derivatives **8**-**10**; and Group 3 was composed of amino derivatives or carbamates **11**-**14**. The compounds showed a wide range of biological activities and some interesting structure-activity relationships were observed. All the results are shown in Table 2 (compound **12** did not show any biological activity in any of the assays, therefore it is not included in Table 2).

#### 2.3.1. Inhibition of photosynthetic electron transport (PET) in spinach chloroplasts

The majority of the studied compounds inhibited PET in spinach chloroplasts, as shown in Table 2. Four compounds showed high inhibitory IC_50_ values: 1.0 µmol/L (**14**), 1.6 µmol/L (**13**), 1.6 µmol/L (**3**) and 2.7 µmol/L (**1**), which was comparable with the standard DCMU (IC_50_ = 1.9 µmol/L). The activity of the rest of the studied compounds was moderate or low relative to the standard. PET inhibition by **8** or **9** could not be determined due to precipitation of the compounds during the experiments. With respect to these small but closed specifically substituted groups of compounds some structure-activity relationships (SAR) can be proposed.

Within Group 1 (salicylanilides; compounds **1**-**5**), the highest PET-inhibiting activity was shown by compounds **3** (2-OMe) and **1** (2-Me). Within the series of 2-alkoxy substituted compounds (**3**-**5**), their activity decreased with increasing lipophilicity (log *k*) and higher substituent bulkiness (substituent bulkiness (MR) and Hammett’s parameter (σ) are described in Table 1 [47]). It is probable that σ (especially the electron-donating effect) is also important for explaining biological activity and searching for structure-activity relationships within a series of compounds [47]. The inhibitory activity of compound **2** was two orders lower (IC_50_ = 331.4 μmol/L) than the activity of compounds **1** and **3**, with comparable log *k* values (0.6661 and 0.6770, respectively). Therefore, when compounds **1** and **2** are considered, similar statements can be made: higher lipophilicity and substituent bulkiness decreases the inhibition of PET. 

Generally, Group 2 (4-nitrobenzencarboxamides) showed only slight PET inhibition caused by the low solubility of compounds **8** and **9** in the testing medium. Compound **10** alone showed medium PET inhibiting activity.

Group 3 (4-aminobenzencarboxamide derivatives) showed very interesting PET activity. Compound **11** with a primary amino moiety possessed very low activity, while substitution of hydrogen in the 4-NH_2_ group of (**11**) by methyl acetate generating compounds **13** and **14** (carbamoylphenylcarbamates), resulted in a strong increase in the inhibitory activity of PET. This finding underlined the importance of the amide group, which can interact with amino acid residues or peptide bonds of proteins situated in photosystems, which can result in PET inhibition. It can be stated that an increase in lipophilicity positively influences PET-inhibiting activity, contrary to Group 1 compounds. Thus, it can be concluded that PET-inhibiting activity is increased by the electron-withdrawing effect and bulkiness of substituents in the anilide part of the molecule.

**Figure 2:**
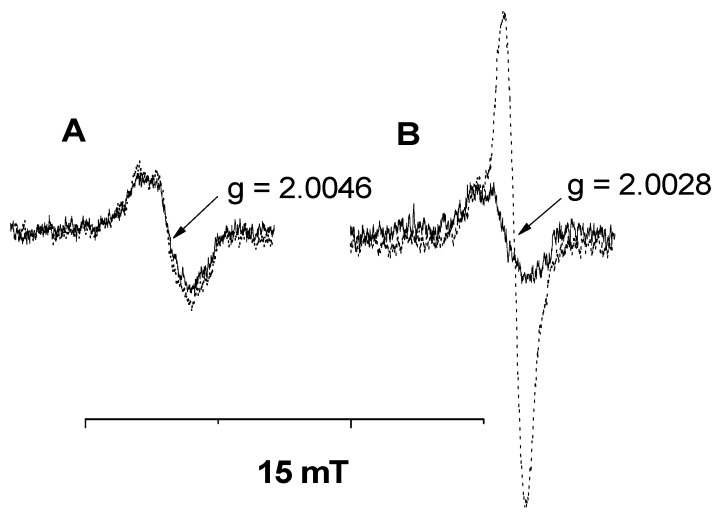
EPR spectra of control chloroplasts **(A)** and chloroplasts treated with 0.05 M of compound **3 (B)**. Full lines were recorded in darkness. Dotted lines were recorded under irradiation (~400 μE/m^2^s PAR) directly in the resonance cavity with a 250 W halogen lamp from 0.5 m distance through a 5 cm water filter.

Experiments were then performed to determine the site of action of the studied compounds in the photosynthetic apparatus. In Figure 2, EPR spectra of untreated chloroplasts and those treated with the very effective PET inhibitor, compound **3** (Table 2), are presented. The EPR spectrum of untreated chloroplasts recorded in the dark consists of the typical signal II_slow_ (*g* = 2.0046, ΔB_pp_= 2 mT, see Figure 2A - full line). An increase of EPR signal intensity in the light represents signal II_very fast_ (*g* = 2.0046, ΔB_pp_= 2 mT; in Figure 2A it is the difference between dotted and full lines). These EPR signals belong to the intermediates D^•^ and Z^•^ respectively, which are tyrosine radicals situated in the 161^st^ positions in D_2_ (for D) and D_1_ (for Z) proteins at the donor side of PS 2 [48,49]. The treatment of chloroplasts with compound **3** evoked a marked increase in the intensity of the EPR signal in the irradiated chloroplasts (Figure 2B, dotted line). On the other hand, in the absence of light, no changes between EPR signal of chloroplasts treated with compound **3** and that of untreated chloroplasts were observed (Figure 2B, full line). The above-mentioned EPR signal (g = 2.0028, ΔB_pp_= 0.8 mT, Figure 2B, dotted line) is denoted as signal I and belongs to the oxidized chlorophyll *a* dimer in the core of PS 1 [50]. The pronounced increase of signal I intensity is caused by an interruption of PET between PS 2 and PS 1. In untreated chloroplasts, after the excitation of chlorophyll *a* dimer (P700) in the core of PS 1 and its subsequent oxidation by the first electron acceptor of PS 1, P700 is reduced by electrons supplied from PS 2, so the intensity of EPR signal I is very small. On the other hand, the disruption of PET between PS 2 and PS 1 results in a great increase of signal I intensity.

To further determine the site of action of the studied compounds, experiments with 1,5-diphenylcarbazide (DPC) were performed. DPC is an artificial electron donor for PS 2 with a known site of action in the Z^•^/D^•^ intermediate [27]. The site of action of the artificial electron acceptor 2,6-dichlorophenol-indophenol (DCPIP) used for monitoring the Hill reaction is plastoquinone on the acceptor side of PS 2 [51]. Thus, if the part of the photosynthetic electron transport chain between manganese cluster and Z/D is inhibited, then DPC will restore PET through PS 2. It was found that after the addition of DPC (2 mmol/L) to chloroplasts treated with the most active compounds **3** and **13**, PET through PS 2 was not restored. On the other hand, after the addition of 40 μmol/L of DCPIPH_2_, which is an artificial electron donor of PS 1 and which has a site of action in plastocyanin on the donor side of PS 1 [51], completely restored PET through PS 1. Based on the above-mentioned experimental results, we suggest that the site of action of the studied compounds could be in Q_B_, which is the second quinone acceptor situated on the oxidizing (acceptor) site of PS 2.

#### 2.3.2. *In vitro* anti-fungal and anti-bacterial susceptibility testing

All discussed above, compounds were tested for their *in vitro* anti-fungal and anti-bacterial activity, although only the salicylanilides **1**-**5** (Group 1), showed some broad spectrum activity. The rest of the compounds did not show any significant activity, potentially due to low solubility in the testing medium and their precipitation during the incubation period. The results are shown in Table 2. Among a number of fungi strains, *Absidia corymbifera*, as a common human pathogen causing pulmonary, rhinocerebral, disseminated, CNS or cutaneous infections and *Trichophyton mentagrophytes* as an example of a dermatophyte carrying a variety of cutaneous infections were selected.

All the compounds in Group 1 showed similar structure-activity relationships with respect to anti-fungal and anti-bacterial activity. Compounds **2** (2,6-CH_3_), **3** (2-OCH_3_) and **1** (2-CH_3_) exhibited low biological activity. Compounds with the longer alkoxy chain **4** (2-OC_2_H_5_) and **5** (2-OC_3_H_7_) showed higher anti-fungal and anti-bacterial activity. With respect to these specific alkyl/alkoxy substituted compounds it can be assumed that a lipophilicity increase and a bulkier substituent seem to be important factors for increasing both of these previously described activities. 2-Hydroxy-*N*-(2-propoxyphenyl)benzamide (**5**) showed similar chemotherapeutic activity against all tested fungal and bacterial strains. Notably, compound **5** demonstrated much higher activity against *Absidia corymbifera* than the standard fluconazole and also higher activity against methicillin resistant *Staphylococcus aureus*, and *Staphylococcus epidermidis* than the common clinically used antibiotics penicillin G and ciprofloxacin.

#### 2.3.3. *In vitro* antimycobacterial evaluation

Although all the compounds discussed above were evaluated for their *in vitro* anti-mycobacterial activity against atypical mycobacterial strains, only compound **1** showed moderate activity, therefore no thorough structure-activity relationships could be established. According to the results presented in Table 2, it can be concluded that compound **1** exhibited activity against *M. avium paratuberculosis* and that its efficacy was comparable with that of the standard, isoniazid.

#### 2.3.4. *In vitro* anti-proliferative activity

Carbonyl-containing moieties are potential iron chelating agents, with many demonstrating anti-proliferative activity due to their ability to bind cellular iron, which is required for proliferation [52]. Therefore, the anti-proliferative activity of compounds **1**-**5** was examined against the human SK-N-MC neuroepithelioma cell line to determine if there were any undesired cytotoxic side-effects. The SK-N-MC cell line was chosen as the effect of iron chelators on the proliferation of these cells has been extensively examined [53,54]. The anti-proliferative activity of the evaluated compounds was assessed in comparison to the well-known clinically used iron chelator, desferrioxamine (DFO), and the highly cytotoxic chelator, di-2-pyridylketone 4,4-dimethyl-3-thiosemicarbazone (Dp44mT) [52].

All tested compounds **1**-**5** demonstrated poor anti-proliferative effects against the SK-N-MC cell line, with IC_50_ values >6.25 µmol/L. As expected from our previous studies [53,54], the iron chelator, DFO, demonstrated poor anti-cancer activity, with an IC_50_ value of 17.07 ± 3.77 µmol/L, while the cytotoxic chelator, Dp44mT (IC_50_ = 0.01 ± 0.01 µmol/L), showed potent anti-proliferative effects. These results suggest that the poor anti-proliferative activity of the anilides **1**-**5** will lead to limited cytotoxicity if ever used as anti-fungal, anti-bacterial or anti-mycobacterial agents.

## 3. Experimental

### 3.1. General

All reagents were purchased from Aldrich. Kieselgel 60, 0.040-0.063 mm (Merck, Darmstadt, Germany) was used for column chromatography. TLC experiments were performed on alumina-backed silica gel 40 F254 plates (Merck, Darmstadt, Germany). The plates were illuminated under UV (254 nm) and evaluated in iodine vapour. The melting points were determined on Boetius PHMK 05 (VEB Kombinat Nagema, Radebeul, Germany) and are uncorrected. The purity of the final compounds was checked by a HPLC separation module (Waters Alliance 2695 XE, Waters Corp., Milford, MA, USA). The detection wavelength of 210 nm was chosen. The peaks in the chromatogram of the solvent (blank) were deducted from the peaks in the chromatogram of the sample solution. The purity of individual compounds was determined from the area peaks in the chromatogram of the sample solution. UV spectra (λ, nm) were determined on a Waters Photodiode Array Detector 2996 (Waters Corp., Milford, MA, USA) in *ca.* 6 × 10^-4^ M methanolic solution and log ε (the logarithm of molar absorption coefficient ε) was calculated for the absolute maximum λ_max_ of individual target compounds. Infrared (IR) spectra were recorded on a Smart MIRacle™ ATR ZnSe for Nicolet™ Impact 410 FT-IR Spectrometer (Thermo Scientific, USA). The spectra were obtained by accumulation of 256 scans with 2 cm^-1^ resolution in the region of 4000-600 cm^-1^. All ^1^H and ^13^C NMR spectra were recorded on a Bruker Avance-500 FT-NMR spectrometer (500 MHz for ^1^H and 125 MHz for ^13^C, Bruker Comp., Karlsruhe, Germany). Chemicals shifts are reported in ppm (δ) using internal Si(CH_3_)_4_ as the reference, with diffuse, easily exchangeable signals being omitted. Mass spectra were measured using a LTQ Orbitrap Hybrid Mass Spectrometer (Thermo Electron Corporation, USA) with direct injection into an APCI source (400 °C) in the positive mode.

### 3.2. Synthesis

#### 3.2.1. General procedure for synthesis of carboxamide derivatives **1**-**5**

2-(Chlorocarbonyl)phenyl acetate (0.014 mol) dissolved in dry acetone (50 mL) was added drop-wise to a stirred solution of the corresponding substituted aniline (0.02 mmol) in dry pyridine (50 mL) kept at room temperature. After the addition was completed, stirring was continued for another 1 h. The reaction mixture was then poured into cold water (100 mL). The mixture was extracted with Et_2_O, dried over anhydrous MgSO_4_, filtered and the solvent removed *in vacuo*. The crude acetylsalicylanilide was dissolved in a 1 M solution of KOH stirred at 50 °C until clarification. The mixture was then neutralized using 1 M HCl. The crude amide was collected and recrystallized from aqueous ethanol and dried.

*2-Hydroxy-N-(2-methylphenyl)benzamide* (**1**). Yield 35%; m.p. 153-155 °C (m.p. 145-147°C [55]); HPLC purity 99.41%; UV (nm), λ_max_/log ε: 271.1/3.42; IR (Zn/Se ATR, cm^-1^): 3324 (N-H), 1611 (C=O), 3741 (O-H), 1542 (C=C); ^1^H-NMR (DMSO-*d*_6_), δ: 12.07 (bs, 1H, OH), 10.31 (bs, 1H, NH), 8.04 (dd, 1H, *J* = 7.9 Hz, *J* = 1.8 Hz, Ar), 7.80 (d, 1H, *J* = 7.9 Hz, Ar), 7.50-7.40 (m, 1H, Ar), 7.28 (d, 1H, *J* = 7.4 Hz, Ar), 7.23 (ddd, 1H, *J* = 7.6 Hz, *J* = 7.6 Hz, *J* = 1.5 Hz, Ar), 7.13 (ddd, 1H, *J* = 7.6 Hz, *J* = 7.6 Hz, *J* = 1.5 Hz, Ar), 7.06-6.94 (m, 2H, Ar), 2.28 (s, 3H, CH_3_); ^13^C-NMR (DMSO-*d*_6_), δ: 165.62, 158.08, 136.16, 133.65, 130.66, 130.30, 129.51, 126.25, 125.14, 123.99, 119.26, 117.20, 117.15, 17.71; HR-MS: for C_14_H_14_O_2_N [M+H]^+^ calculated 228.098 m/z, found 228.0892 m/z.

*N-(2,6-Dimethylphenyl)-2-hydroxybenzamide* (**2**). Yield 37%; m.p. 137-140 °C; HPLC purity 99.73%; UV (nm), λ_max_/log ε: 237.9/3.45; IR (Zn/Se ATR, cm^-1^): 3278 (N-H), 1602 (C=O), 1538 (C=C); ^1^H-NMR (DMSO-*d*_6_), δ: 12.34 (bs, 1H, OH), 10.07 (s, 1H, NH), 8.05 (dd, 1H, *J* = 8.2 Hz, *J* = 1.8 Hz, Ar), 7.51-7.43 (m, 1H, Ar), 7.22-7.10 (m, 3H, Ar), 7.02-6.92 (m, 2H, Ar), 2.19 (s, 6H, CH_3_); ^13^C-NMR (DMSO-*d*_6_), δ: 167.68, 160.06, 135.48, 134.28, 133.95, 128.14, 127.79, 127.00, 118.81, 117.45, 115.38, 17.97; HR-MS: for C_15_H_16_O_2_N [M+H]^+^ calculated 242.118 m/z, found 242.1084 m/z.

*2-Hydroxy-N-(2-methoxyphenyl)benzamide* (**3**). Yield 39%; m.p. 112-115 °C (m.p. 115-117 °C [55]); HPLC purity 99.92%; UV (nm), λ_max_/log ε: 298.4/3.49; IR (Zn/Se ATR, cm^-1^): 3288 (N-H), 1600 (C=O), 1228 (C-O), 1547 (C=C); ^1^H-NMR (DMSO-*d*_6_), δ: 11.70 (bs, 1H, OH), 10.81 (bs, 1H, NH), 8.43-8.36 (m, 1H, Ar), 8.03 (dd, 1H, *J* = 7.9 Hz, *J* = 1.8 Hz, Ar), 7.45-8.38 (m, 1H, Ar), 7.12-7.07 (m, 2H, Ar), 7.02 (dd, 1H, *J* = 8.2 Hz, *J* = 0.9 Hz, Ar), 7.01-6.93 (m, 2H, Ar), 3.89 (s, 1H, CH_3_); ^13^C-NMR (DMSO-*d*_6_), δ: 163.43, 156.28, 148.69, 133.32, 130.67, 127.84, 123.89, 120.57, 120.30, 119.59, 118.74, 116.90, 110.96, 55.99; HR-MS: for C_14_H_14_O_3_N [M+H]^+^ calculated 244.098 m/z, found 244.0980 m/z.

*N-(2-Ethoxyphenyl)-2-hydroxybenzamide* (**4**). Yield 35%; m.p. 105-107 °C; HPLC purity 99.94%; UV (nm), λ_max_/log ε: 299.6/3.48; IR (Zn/Se ATR, cm^-1^): 3418 (N-H), 1604 (C=O), 3736 (O-H), 1216 (C-O); ^1^H-NMR (DMSO-*d*_6_), δ: 11.54 (bs, 1H, OH), 10.93 (bs, 1H, NH), 8.50-8.42 (m, 1H, Ar), 8.04 (dd, 1H, *J* = 7.9 Hz, *J* = 1.8 Hz, Ar), 7.45-8.39 (m, 1H, Ar), 7.10-7.02 (m, 3H, Ar), 7.01-6.97 (m, 1H, Ar), 6.97-6.92 (m, 1H, Ar), 4.12 (q, 2H, *J* = 6.9 Hz, CH_2_), 1.44 (t, 3H, *J* = 6.9 Hz, CH_3_); ^13^C-NMR (DMSO-*d*_6_), δ: 163.01, 156.02, 147.65, 133.25, 130.87, 128.32, 123.57, 120.54, 119.70, 119.65, 118.98, 116.85, 111.79, 64.08, 14.75; HR-MS: for C_15_H_16_O_3_N [M+H]^+^ calculated 258.118 m/z, found 258.0889 m/z.

*2-Hydroxy-N-(2-propoxyphenyl)benzamide* (**5**). Yield 31%; m.p. 92-95 °C; HPLC purity 99.98%; UV (nm), λ_max_/log ε: 299.6/3.39; IR (Zn/Se ATR, cm^-1^): 3281 (N-H), 1598 (C=O), 1544 (C=C), 1241 (C-O); ^1^H-NMR (DMSO-*d*_6_), δ: 11.49 (bs, 1H, OH), 10.86 (bs, 1H, NH), 8.49-8.44 (m, 1H, Ar), 8.04 (dd, 1H, *J* = 7.9 Hz, *J* = 1.8 Hz, Ar), 7.44-7.37 (m, 1H, Ar), 7.11-7.02 (m, 3H, Ar), 7.02-6.96 (m, 1H, Ar), 6.96-6.92 (m, 1H, Ar), 4.02 (t, 2H, *J* = 6.5 Hz, CH_2_CH_2_), 1.84 (q, 2H, *J* = 7.0 Hz CH_2_CH_2_), 1.04 (t, 3H, *J* = 7.3 Hz, CH_3_); ^13^C-NMR (DMSO-*d*_6_), δ: 163.07, 156.06, 147.79, 133.25, 130.88, 128.17, 123.64, 120.41, 119.87, 119.64, 118.95, 116.86, 111.60, 69.82, 22.16, 10.51; HR-MS: for C_16_H_18_O_3_N [M+H]^+^ calculated 272.128 m/z, found 272.1280 m/z.

*4-Nitrobenzoic acid* (**6**). 4-Nitrobenzaldehyde (0.15 mol) in acetone (600 mL) and 10% aqueous solution of KMnO_4_ (400 mL) was refluxed for 90 min. The mixture was then filtered and the resulting colourless solution was concentrated *in vacuo* to 300 mL. The solution was acidified by 35% HCl. The crude product was filtered, washed by water, recrystallized from aqueous ethanol and dried. Yield: 75%; m.p. 237-240 °C.

*4-Nitrobenzoyl chloride* (**7**). Acid **6** (0.05 mol) and thionyl chloride (0.15 mmol) was refluxed for about 1 h. The excess of thionyl chloride was removed by repeated evaporation with dry toluene *in vacuo*. The crude product was recrystallized form anhydrous petroleum ether. Yield: 100%; m.p. 75 °C.

#### 3.2.2. General procedure for synthesis of carboxamide derivatives **8**-**10**

The acyl chloride **7** (0.014 mol) dissolved in dry acetone (50 mL) was added drop-wise to a stirred solution of the corresponding substituted amine (0.02 mmol) in 50 mL of dry pyridine kept at the room temperature. After the addition was completed, stirring continued for another 1 h. The reaction mixture was then poured into 100 mL of cold water and the crude amide was collected and recrystallized from aqueous ethanol and dried.

*N-(2-Chlorophenyl)-4-nitrobenzamide* (**8**). Yield 75%; m.p. 165-167 °C (m.p. 157-158 °C [56]); HPLC purity 99.73%; UV (nm), λ_max_/log ε: 264.0/3.44; IR (Zn/Se ATR, cm^-1^): 3299 (N-H), 1653 (C=O), 680 (C-Cl), 1514, 1342 (NO_2_), 1584 (C=C); ^1^H-NMR (DMSO-*d*_6_), δ: 10.45 (bs, 1H, NH), 8.39 (d, 2H, *J* = 8.9 Hz, Ar), 8.22 (d, 2H, *J* = 8.9 Hz, Ar), 7.63-7.55 (m, 2H, Ar), 7.42 (ddd, 1H, *J* = 7.7 Hz, *J* = 7.7 Hz, *J* = 1.5 Hz, Ar), 7.34 (ddd, 1H, *J* = 7.7 Hz, *J* = 7.7 Hz, *J* = 1.7 Hz, Ar); ^13^C-NMR (DMSO-*d*_6_), δ: 163.91, 149.33, 139.57, 134.53, 129.69, 129.62, 129.20, 128.62, 127.94, 127.56, 123.66; HR-MS: for C_13_H_10_O_3_N_2_Cl [M+H]^+^ calculated 277.038 m/z, found 277.0354 m/z.

*N-(2-Chlorobenzyl)-4-nitrobenzamide* (**9**). Yield 78%; m.p. 178 °C; HPLC purity 99.81%; UV (nm), λ_max_/log ε: 265.2/3.41; ^1^H-NMR (DMSO-*d*_6_), δ: 9.39 (bt, 1H, *J* = 5.6 Hz, NH), 8.34 (d, 2H, *J* = 8.9 Hz, Ar), 8.14 (d, 2H, *J* = 8.9 Hz, Ar), 7.50-7.43 (m, 1H, Ar), 7.43-7.37 (m, 1H, Ar), 7.37-7.28 (m, 2H, Ar), 4.58 (d, 2H, *J* = 5.6 Hz, CH_2_); ^13^C-NMR (DMSO-*d*_6_), δ: 164.82, 149.10, 139.67, 135.82, 132.06, 129.16, 128.87, 128.83, 128.72, 127.20, 123.55, 40.81; HR-MS: for C_14_H_12_O_3_N_2_Cl [M+H]^+^ calculated 291.058 m/z, found 291.0530 m/z.

*N-[2-Chloro-5-(trifluoromethyl)phenyl]-4-nitrobenzamide* (**10**). Yield 79%; m.p. 182 °C; HPLC purity 99.95%; UV (nm), λ_max_/log ε: 268.7/3.42; ^1^H-NMR (DMSO-*d*_6_), δ: 10.66 (bs, 1H, NH), 8.40 (d, 2H, *J* = 8.9 Hz, Ar), 8.22 (d, 2H, *J* = 8.9 Hz, Ar), 8.05 (d, 1H, *J* = 2.0 Hz, Ar), 7.85 (d, 1H, *J* = 8.4 Hz, Ar), 7.70 (dd, 1H, *J* = 8.4 Hz, *J* = 2.0 Hz, Ar); ^13^C-NMR (DMSO-*d*_6_), δ: 164.19, 149.47, 139.18, 135.56, 133.76, 130.93, 129.33, 128.17 (q, 1C, ^2^*J*_FC_ = 32.8 Hz), 124.97 (q, 1C, ^3-1^*J*_FC_ = 3.8 Hz), 124.33 (q, 1C, ^3-2^*J*_FC_ = 3.8 Hz), 123.67, 123.51 (q, 1C, ^1^*J*_FC_ = 272.5 Hz); HR-MS: for C_14_H_9_O_3_N_2_ClF_3_ [M+H]^+^ calculated 345.028 m/z, found 345.0238 m/z.

*4-Amino-N-(2-chlorobenzyl)benzamide* (**11**). Nitrobenzamide **9** (0.01 mol) was dissolved in MeOH (50 mL) and suspension of activated Ra-Ni (3 g) [43] was added. The suspension was mixed and vigorously stirred for 24 h under hydrogen atmosphere from balloon. Then the reaction mixture was filtered and evaporated *in vacuo*. The crude product was recrystallized from aqueous ethanol and dried. Yield 85%; m.p. 137 °C; HPLC purity 99.51%; UV (nm), λ_max_/log ε: 279.4/3.38; ^1^H-NMR (DMSO-*d*_6_), δ: 8.56 (bt, 1H, *J* = 5.8 Hz, NH), 7.64 (d, 2H, *J* = 8.6 Hz, Ar), 7.47-7.39 (m, 1H, Ar), 7.35-7.22 (m, 3H, Ar), 6.56 (d, 2H, *J* = 8.6 Hz, -CH=C(NH_2_)-CH=), 5.64 (bs, 2H, NH_2_), 4.48 (d, 2H, *J* = 5.8 Hz, CH_2_); ^13^C-NMR (DMSO-*d*_6_), δ: 166.39, 151.79, 136.98, 131.74, 128.96, 128.82, 128.35, 128.26, 127.03, 120.67, 112.53, 40.22; HR-MS: for C_14_H_14_ON_2_Cl [M+H]^+^ calculated 261.078 m/z, found 261.0789 m/z.

*4-Amino-N-[2-chloro-5-(trifluoromethyl)phenyl]benzamide* (**12**). Nitrobenzamide **10** (0.01 mol) was dissolved in mixture MeOH:THF 1:1 (50 mL) and suspension of activated Ra-Ni (3 g) [44] was added. Hydrogenation was realized in an autoclave under 65-75 psi for 24 h. Then the reaction mixture was filtered and evaporated *in vacuo*. The crude product was recrystallized from aqueous ethanol and dried. Yield 80%; m.p. 151 °C; ^1^H-NMR (DMSO-*d*_6_), δ: 10.15 (bs, 1H, NH), 8.09 (d, 1H, *J* = 2.0 Hz, Ar), 8.07 (d, 1H, *J* = 8.4 Hz, Ar), 8.05 (dd, 1H, *J* = 8.4 Hz, *J*=2.0 Hz, Ar), 7.79 (d, 2H, *J* = 8.6 Hz, Ar), 6.64 (d, 2H, *J*=8.6 Hz, -CH=C(NH_2_)-CH=), 5.86 (bs, 2H, NH_2_); ^13^C-NMR (DMSO-*d*_6_), δ: 165.66, 152.50, 136.67, 132.25, 130.59, 129.56, 129.35 (q, 1C, ^2^*J*_FC_ = 32.04 Hz), 125.34 (q, 1C, ^1^*J*_FC_ = 272.53 Hz), 124.76 (d, 1C, ^3-1^*J*_FC_ = 3.42 Hz), 123.47 (d, 1C, ^3-2^*J*_FC_ = 3.62 Hz), 120.38, 112.58.

*Methyl 4-(2-chlorobenzylcarbamoyl)phenylcarbamate* (**13**). Aminobenzamide **11** (0.01 mol) was dissolved in acetone (30 mL) and K_2_CO_3_ (0.015 mol) was added. Then methyl chloroformiate (0.015 mol) was added drop-wise. The mixture was refluxed for 24 h. After cooling the solid was filtered and solution was evaporated *in vacuo*. The crude product was washed by water and crystallized from EtOH. Yield 90%; m.p. 205 °C; HPLC purity 97.24%; UV (nm), λ_max_/log ε: 264.6/3.39; IR (Zn/Se ATR, cm^-1^): 3302 (N-H), 3345 (OCON-H), 1704 (OC=O), 1630 (C=O), 1527 (C=C), 1229 (C-O), 733 (C-Cl); ^1^H-NMR (DMSO-*d*_6_), δ: 9.93 (bs, 1H, OCONH), 8.89 (bt, 1H, NH, *J* = 5.8 Hz), 7.86 (d, 2H, *J* = 8.7 Hz, Ar), 7.55 (d, 2H, *J* = 8.7 Hz, Ar), 7.45 (dd, 1H, *J* = 7.4 Hz, *J* = 1.6 Hz, Ar), 7.38-7.24 (m, 3H, Ar), 4.53 (d, 2H, *J* = 5.8 Hz, CH_2_), 3.69 (s, 3H, CH_3_); ^13^C-NMR (DMSO-*d*_6_), δ: 165.90, 153.84, 142.02, 136.50, 131.85, 129.04, 128.50, 128.44, 128.23, 127.75, 127.11, 117.22, 51.77, 40.42; HR-MS: for C_16_H_16_O_3_N_2_Cl [M+H]^+^ calculated 319.088 m/z, found 319.0844 m/z.

*Methyl 4-[2-chloro-5-(trifluoromethyl)phenylcarbamoyl]phenylcarbamate* (**14**). Aminobenzamide **12** (0.01 mol) was dissolved in acetone (30 mL), and K_2_CO_3_ (0.015 mol) was added. Then methyl chloroformiate (0.015 mol) was added dropwise. The mixture was refluxed for 24 h. After cooling the solid was filtered and solution was evaporated *in vacuo*. The crude product was washed by water and crystallized from hexane. Yield 76%; Mp. 173°C; HPLC purity 93.33%; UV (nm), λ_max_/log ε: 278.2/3.43; IR (Zn/Se ATR, cm^-1^): 3296 (N-H), 1714 (OC=O), 1653 (C=O), 1532 (C=C), 1257 (C-O), 1171, 1117 (C-F), 662 (C-Cl); ^1^H-NMR (DMSO-*d*_6_), δ: 10.08 (s, 1H, NH), 10.03 (s, 1H, OCONH), 8.08-8.01 (m, 1H, Ar), 7.96 (d, 2H, *J* = 8.4 Hz, Ar), 7.81 (d, 1H, *J* = 8.4 Hz, Ar), 7.67-7.62 (m, 1H, Ar), 7.62 (d, 2H, *J* = 8.8 Hz, Ar), 3.71 (s, 3H, CH_3_); ^13^C-NMR (DMSO-*d*_6_), δ: 164.93, 153.84, 142.78, 136.18, 133.14, 130.76, 128.87, 128.04 (q, 1C, ^2^*J*_FC_ = 32.6 Hz), 126.99, 124.27 (q, 1C, ^3-1^*J*_FC_ = 3.8 Hz), 123.63 (q, 1C, ^1^*J*_FC_ = 272.48 Hz), 123.49 (q, 1C, ^3-2^*J*_FC_ = 3.8 Hz), 117.30, 51.85; HR-MS: for C_16_H_13_O_3_N_2_ClF_3_ [M+H]^+^ calculated 373.738 m/z, found 373.0562 m/z.

### 3.3. Lipophilicity determination by HPLC (capacity factor k/calculated log k)

A Waters Alliance 2695 XE HPLC separation module and a Waters Photodiode Array Detector 2996 (Waters Corp., Milford, MA, USA) were used. A Symmetry^®^ C_18_ 5 μm, 4.6 × 250 mm, Part No. WAT054275 (Waters Corp., Milford, MA, USA) chromatographic column was used. The HPLC separation process was monitored by Empower™ 2 Chromatography Data Software, Waters 2009 (Waters Corp., Milford, MA, USA). A mixture of MeOH p.a. (70%) and H_2_O-HPLC – Mili-Q Grade (30%) was used as a mobile phase. The total flow of the column was 1.0 mL/min, injection volume 30 μL, column temperature 30 °C and sample temperature 10°C. The detection wavelength of 210 nm was chosen. The KI methanolic solution was used for the dead time (t_D_) determination. Retention times (t_R_) were measured in minutes. The capacity factors *k* were calculated using the Empower™ 2 Chromatography Data Software according to formula *k* = (t_R_ - t_D_)/t_D_, where t_R_ is the retention time of the solute, whereas t_D_ denotes the dead time obtained using an unretained analyte. Log *k*, calculated from the capacity factor *k*, is used as the lipophilicity index converted to log *P* scale. The log *k* values of the individual compounds are shown in Table 1.

### 3.4. Lipophilicity calculations

Log *P*, *i.e.* the logarithm of the partition coefficient for *n-*octanol/water, was calculated using the programs CS ChemOffice Ultra ver. 10.0 (CambridgeSoft, Cambridge, MA, USA) and ACD/LogP ver. 1.0 (Advanced Chemistry Development Inc., Toronto, Canada). Clog *P* values (the logarithm of *n*-octanol/water partition coefficient based on established chemical interactions) were generated by means of CS ChemOffice Ultra ver. 10.0 (CambridgeSoft, Cambridge, MA, USA) software. The results are shown in Table 1.

### 3.5. Study of inhibition photosynthetic electron transport (PET) in spinach chloroplasts

Chloroplasts were prepared from spinach (*Spinacia oleracea* L.) according to Masarovicova and Kralova [57]. The inhibition of photosynthetic electron transport (PET) in spinach chloroplasts was determined spectrophotometrically (Genesys 6, Thermo Scientific, USA), using an artificial electron acceptor 2,6-dichlorophenol-indophenol (DCIPP) according to Kralova *et al*. [58], and the rate of photosynthetic electron transport was monitored as a photoreduction of DCPIP. The measurements were carried out in phosphate buffer (0.02 mol/L, pH 7.2) containing sucrose (0.4 mol/L), MgCl_2_ (0.005 mol/L) and NaCl (0.015 mol/L). The chlorophyll content was 30 mg/L in these experiments and the samples were irradiated (~100 W/m^2^) from 10 cm distance with a halogen lamp (250 W) using a 4 cm water filter to prevent warming of the samples (suspension temperature 22 °C). The studied compounds were dissolved in DMSO due to their limited water solubility. The applied DMSO concentration (up to 4%) did not affect the photochemical activity in spinach chloroplasts. The inhibitory efficiency of the studied compounds was expressed by IC_50_ values, *i.e.* by molar concentration of the compounds causing 50% decrease in the oxygen evolution rate relative to the untreated control. The comparable IC_50_ value for a selective herbicide 3-(3,4-dichlorophenyl)-1,1-dimethylurea, DCMU (Diurone^®^) was about 1.9 μmol/L [59]. The results are summarized in Table 2.

EPR spectra were registered by the equipment ERS 230 (ZWG, Acad. Sci., Berlin, Germany), which operates in X-band (~9.3 GHz), with modulation amplitude 0.5 mT and microwave power 5 mW at room temperature. The samples containing chlorophyll (3.2 g/L) were measured in a flat quartz cell and their irradiation (~400 μE/m^2^s PAR) was carried out directly in the resonance cavity with a 250 W halogen lamp from 0.5 m distance through 5 cm water filter.

### 3.6. In vitro anti-fungal susceptibility testing

The broth microdilution test [60] was used for the assessment of *in vitro* anti-fungal activity of the synthesized compounds against *Absidia corymbifera* 272 (AC) and *Trichophyton mentagrophytes* 445 (TM). Fluconazole (FLU) was used as the standard since it is a clinically used anti-mycotic drug. The procedure was performed with a two-fold dilution of the compounds in RPMI 1640 (Sevapharma a.s., Prague, Czech Republic) buffered to pH 7.0 with 0.165 mol of 3-morpholino-propane-1-sulfonic acid (MOPS, Sigma, Germany). The final concentrations of the compounds ranged from 500 to 0.975 μmol. Drug–free controls were included. The minimum inhibitory concentration (MIC) determination was performed according to the Clinical and Laboratory Standards Institute (M38-A) for moulds and is an IC_50_ value. IC_50_ values were defined as a 50% reduction of growth in comparison with the control. The values of the minimum inhibitory concentration (MICs) were determined after 24 and 48 h of static incubation at 35 °C. For *T. mentagrophytes*, the final MICs were determined after 72 and 120 h of incubation. The results are summarized in Table 2.

### 3.7. In vitro anti-bacterial susceptibility testing

The synthesized compounds were evaluated for *in vitro* anti-bacterial activity against methicilin resistant *Staphylococcus aureus* H 5996/08 (MRSA), and *Staphylococcus epidermidis* H 6966/08 (SE). Penicillin G (PEN), and ciprofloxacin (CPF) were used as standards since they are clinically used anti-bacterial drugs. All strains were sub-cultured on nutrient agar (HiMedia) and maintained on the same medium at 4°C. Prior to testing, each strain was passaged onto nutrient agar and bacterial inocula were prepared by suspending a small portion of bacterial colony in sterile 0.85% saline. The cell density was adjusted to 0.5 McFarland units using a densitometer (Densi-La-Meter, PLIVA Lachema Diagnostika). The final inoculum was made by 1:20 dilution of the suspension with the test medium (Mueller-Hinton broth). The compounds were dissolved in DMSO and the anti-bacterial activity was determined using Mueller-Hinton broth (MH broth, HiMedia, pH 7.0 ± 0.2). Controls consisted of MH broth and DMSO alone. The final concentration of DMSO in the MH broth did not exceed 1% (v/v) of the total solution composition. The MIC were defined as 90% inhibition of bacterial growth compared to control and were determined after 24 and 48 h of static incubation at 37 °C. The results are shown in Table 2.

### 3.8. In vitro anti-mycobacterial evaluation

Clinical isolates of *Mycobacterium avium* complex CIT19/06 (MAC), *M. avium paratuberculosis* ATCC19698 (MAP), and *M. kansasii* CIT11/06 (MK) were grown in Middlebrook broth (MB), supplemented with Oleic, Albumin, Dextrose, Catalase supplement (OADC, Becton Dickinson, UK). Identification of these isolates was performed using biochemical and molecular protocols. At log phase growth, culture (10 mL) was centrifuged at 15,000 rpm/20 min using a bench top centrifuge (Model CR 4-12 Jouan Inc., UK). Following removal of the supernatant, the pellet was washed in fresh Middlebrook 7H9GC broth and re-suspended in fresh supplemented MB (10 mL). The turbidity was adjusted to match McFarland standard No. 1 (3 × 10^8^ cfu) with MB broth. A further 1:20 dilution of the culture was then performed in MB broth.

The anti-microbial susceptibility of all three mycobacterial species was investigated in a 96-well plate format. In these experiments, sterile deionised water (300 µL) was added to all outer-perimeter wells of the plates to minimize evaporation of the medium in the test wells during incubation. Each evaluated compound (100 µL) was incubated with each of the mycobacterial species (100 µL). Dilutions of each compound were prepared in duplicate. For all synthesized compounds, final concentrations ranged from 1,000 µg/mL to 8 µg/mL. All compounds were prepared in DMSO and subsequent dilutions were made in supplemented MB. The plates were sealed with parafilm and incubated at 37°C, for 5 days in the case of *M. kansasii* and *M. avium complex* and 7 days in the case of *M. avium paratuberculosis*. Following incubation, a 10% addition of alamarBlue (AbD Serotec) was mixed into each well and readings at 570 nm and 600 nm were taken, initially for background subtraction and subsequently after 24 h re-incubation. The background subtraction is necessary for strongly coloured compounds, where the colour may interfere with the interpretation of any colour change [61]. For non-interfering compounds, a blue colour in the well was interpreted as an absence of growth and a pink colour was scored as growth. The MIC was initially defined as the lowest concentration which prevented a visual colour change from blue to pink. Isoniazid (INH) was used as the standard as it is a clinically used anti-mycobacterial drug. The results are shown in Table 2. The MIC for mycobacteria was defined as a 90% or greater (IC_90_) reduction of growth in comparison with the control. The MIC/IC_90_ value is routinely and widely used in bacterial assays and is a standard detection limit according to the Clinical and Laboratory Standards Institute (CLSI, www.clsi.org/).

### 3.9. Cell culture and in vitro antiproliferative activity

The human SK-N-MC neuroepithelioma cell type was obtained from the American Type Culture Collection (Manassas, VA, USA) and was cultured in minimum essential medium (MEM; Gibco, Melbourne, Australia) containing 10% (v/v) FBS, 1.0 mM sodium pyruvate (Gibco), 1% (v/v) non-essential amino acids (Gibco), 2 mM L-glutamine (Gibco), 100 U/mL penicillin (Gibco), streptomycin (Gibco) and 0.28 μg/mL fungizone (Squibb Pharmaceuticals, Montreal, Canada). Cells were cultured under standard conditions at 37°C, in a humidified atmosphere at 5% CO_2_.

The effect of the compounds on cellular proliferation was determined by the MTT [1-(4,5-dimethylthiazol-2-yl)-2,5-diphenyl tetrazolium] assay using standard techniques [53,54]. Previous studies have demonstrated that MTT reduction is proportional to viable cell counts using SK-N-MC cells [62]. The SK-N-MC cells were seeded in 96-well microtiter plates at 1.5 × 10^4^ cells/well in medium containing human diferric transferrin (Tf) at 1.25 μmol/L ([Fe] = 2.5 μmol/L) and compounds at a range of concentrations (0-25 μmol/L). Control samples contained medium with Fe-transferrin (1.25 μmol/L) without the compounds. The chelators, DFO and Dp44mT, were also included as internal controls, as their effects are well characterized in this cell line [53,54]. The cells were incubated at 37°C in a humidified atmosphere containing 5% CO_2_ and 95% air for 72 h. After 72 h, 10 µL of MTT solution (stock solution: 5 mg/mL) was added to each well and incubated for 2 h at 37°C. After solubilization of the cells with 100 μL of 10% SDS-50% isobutanol in 0.01 M HCl, the plates were read at 570 nm using a scanning multi-well spectrophotometer. The inhibitory concentration (IC_50_) was defined as the compound concentration necessary to reduce the absorbance to 50% of the untreated control.

## 4. Conclusions

A series of twelve ring-substituted salicylanilides and carbamoylphenylcarbamates were prepared and characterized. Their lipophilicity was determined using a well established RP-HPLC method. The prepared compounds were tested for their ability to inhibit photosynthetic electron transport (PET) in spinach chloroplasts (*Spinacia oleracea* L.) and for their anti-fungal, anti-bacterial, anti-mycobacterial and anti-tumor activity. Four compounds, methyl 4-[2-chloro-5-(trifluoromethyl) phenylcarbamoyl]-phenylcarbamate (**14**), methyl 4-(2-chlorobenzylcarbamoyl)phenylcarbamate (**13**), 2-hydroxy-*N*-(2-methoxyphenyl)benzamide (**3**), and 2-hydroxy-*N*-(2-methylphenyl)benzamide (**1**) showed PET inhibition comparable with or higher than the standard DCMU. The site responsible for the inhibitory action of the studied compounds in the photosynthetic apparatus could be Q_B_, the second quinone acceptor situated on the oxidizing (acceptor) site of PS 2.

Interestingly, 2-hydroxy-*N*-(2-propoxyphenyl)benzamide (**5**) demonstrated higher anti-fungal activity against *Absidia corymbifera* than the standard fluconazole and also higher activity against methicillin-resistant *Staphylococcus aureus* and *Staphylococcus epidermidis* than penicillin G and ciprofloxacin. Compound **1** exhibited activity against *Mycobacterium avium paratuberculosis* that was comparable with the standard isoniazid. None of the agents showed effective anti-tumor activity *in vitro*.

## Figures and Tables

*Reagents and conditions*: a) acetone, pyridine; b) KOH, HCl; c) KMNO_4_, acetone, H_2_O; d), SOCl_2_, toluene; e) H_2_, Ra-Ni, MeOH, THF; f) ClCO_2_Me, acetone, K_2_CO_3_.

**Table 1:** Comparison of the calculated lipophilicities (log *P*/Clog *P*) with the determined log *k* values, Hammett’s parameter (σ) and bulk parameter (MR, reflecting bulkiness).

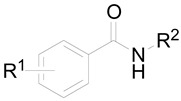							
**Comp.**	**R^1^**	**R^2^**	**log *k***	**log *P*/Clog *P*** ChemOffice	**log *P*** ACD/LogP	**σ** [47]	**MR** [47]
**1**	2-OH	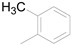	0.6661	2.94 / 3.1212	3.73 ± 0.37	0.10	4.7
**2**	2-OH	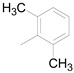	0.6807	3.42 / 2.9702	4.19 ± 0.38	0.20	9.4
**3**	2-OH	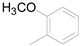	0.6770	2.32 / 2.7576	3.17 ± 0.39	0.00	6.5
**4**	2-OH	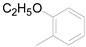	0.7515	2.66 / 3.2866	3.70 ± 0.39	0.02	11.3
**5**	2-OH	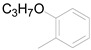	0.8401	3.15 / 3.8156	4.23 ± 0.39	NF	15.9
**8**	4-NO_2_	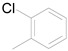	0.6782	3.15 / 2.7782	3.19 ± 0.34	0.67	4.7
**9**	4-NO_2_	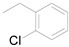	0.6966	2.82 / 3.7170	3.19 ± 0.35	0.67	4.7
**10**	4-NO_2_	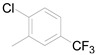	0.8269	4.13 / 3.7726	4.94 ± 0.43	1.10	8.7
**11**	4-NH_2_	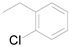	0.5886	2.66 / 2.6350	2.47 ± 0.39	0.67	4.7
**12**	4-NH_2_	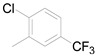	ND	3.52 / 3.0299	4.12 ± 0.42	1.10	8.7
**13**	4-CH_3_OCONH	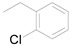	0.6345	2.96 / 3.2510	3.21 ± 0.43	0.67	4.7
**14**	4-CH_3_OCONH	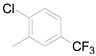	0.7782	3.81 / 3.6459	4.86 ± 0.50	1.10	8.7

NF = not found in literature; ND = not determined/analyzed.

**Table 2:** IC_50_ values of compounds **1**-**11**, **13** and **14** related to photosynthetic electron transport (PET) inhibition in spinach chloroplasts in comparison with the 3-(3,4-dichlorophenyl)-1,1-dimethylurea (DCMU) standard; *in vitro* anti-fungal and anti-bacterial activity (IC_50_/IC_90_) of compounds **1**-**5** compared with the fluconazole (FLU), penicillin G (PEN), ciprofloxacin (CPF) standards and the anti-mycobacterial activity (IC_90_) of compound **1** in comparison with the standard isoniazid (INH).

Comp.	PET inhibition IC_50_ [μmol/L]	MIC [µmol/L]				MIC/IC_90_ [µg/mL]		
		AC*^a^*	TM*^a^*	MRSA*^b^*	SE*^b^*	MAC	MAP	MK
		*24 h* *48 h*	*72 h* *120 h*	*24 h* *48 h*	*24 h* *48 h*			
**1**	2.7	62.50 250	62.50 125	125 250	125 250	250	250	125
**2**	331.4	>250 >250	250 500	>500 >500	>500 >500	NA	NA	NA
**3**	1.6	125 125	125 125	500 >500	>500 >500	NA	NA	NA
**4**	15.0	62.50 125	62.50 62.50	125 125	125 125	NA	NA	NA
**5**	44.8	31.25 31.25	31.25 31.25	31.25 31.25	31.25 31.25	NA	NA	NA
**10**	50.1	NA	NA	NA	NA	NA	NA	NA
**11**	398.4	NA	NA	NA	NA	NA	NA	NA
**13**	1.6	NA	NA	NA	NA	NA	NA	NA
**14**	1.0	NA	NA	NA	NA	NA	NA	NA
**DCMU**	1.9	–	–	–	–	–	–	–
**FLU**	–	>125 >125	1.95 3.91	–	–	–	–	–
**PEN**	–	–	–	125 125	31.62 125			
**CPF**	–	–	–	500 500	250 250			
**INH**	–	–	–	–	–	<10	>250	<10

MIC determination (MIC: minimum inhibitory concentration): *^a^*moulds-fungi (IC_50_ value), *^b^*bacteria (IC_90_ value), NA = no significant activity, AC: *Absidia corymbifera*, TM: *Trichophyton mentagrophytes*, MRSA: methicillin-resistant *Staphylococcus aureus*, SE: *Staphylococcus epidermidis*, MAC: *Mycobacterium avium* complex, MAP: *M. avium paratuberculosis*, MK*: M. kansasii*).
